# Genotypic Diversity of Human Rhinovirus in Children with Pneumonia Before and During the COVID-19 Pandemic in Mexico

**DOI:** 10.3390/pathogens14121236

**Published:** 2025-12-04

**Authors:** Janet Sánchez-Ramos, Miguel Leonardo García-León, Patricia Bautista-Carbajal, Luis Alfonso Salazar-Soto, Daniel E. Noyola, María Susana Juárez-Tobías, Pedro Antonio Martínez-Arce, María Del Carmen Espinosa-Sotero, Verónica Tabla-Orozco, Gerardo Martínez-Aguilar, Fabian Rojas-Larios, Izveidy Mondragón-Salinas, Rosa María Wong-Chew

**Affiliations:** 1 Infectious Diseases Research Laboratory, Research Division, Facultad de Medicina, Universidad Nacional Autónoma de México (UNAM), Mexico City 04510, Mexico; janetbiologia@gmail.com (J.S.-R.);; 2 Center for Health Sciences and Biomedicine Research, Facultad de Medicina, Universidad Autónoma de San Luis Potosí, San Luis Potosí 78210, San Luis Potosí, Mexico; dnoyola@uaslp.mx; 3 Department of Pediatrics, Hospital Regional de Alta Especialidad Dr. Ignacio Morones Prieto, San Luis Potosí 78290, San Luis Potosí, Mexico; 4 Department of Pediatric Infectious Diseases, Antiguo Hospital Civil de Guadalajara Fray Antonio Alcalde, Guadalajara 44200, Jalisco, Mexico; 5 Department of Pediatrics, Hospital General de México Dr. Eduardo Liceaga, Mexico City 06760, Mexico; 6 Emergency Department, Hospital Pediátrico de Coyoacán, Mexico City 04000, Mexico; 7 Hospital Materno Infantil, Durango 34000, Durango, Mexico; uimec@yahoo.es; 8 Facultad de Medicina y Nutrición, Universidad Juárez del estado de Durango, Durango 34076, Durango, Mexico; 9 Hospital Regional Universitario IMSS Bienestar de Colima, Colima 28000, Colima, Mexico; 10 Hospital de las Culturas, San Cristóbal de las Casas 29264, Chiapas, Mexico

**Keywords:** human rhinovirus, children, pneumonia, genotypes

## Abstract

Human rhinovirus (HRV) is one of the most common viral causes of respiratory tract infections worldwide. The COVID-19 pandemic and non-pharmaceutical interventions significantly altered the epidemiology of respiratory viruses. This study compared HRV genotypic diversity in Mexican children with pneumonia before and during the pandemic. A total of 1983 children with pneumonia were included: 1404 pre-pandemic (2010–2013) and 579 pandemic (2021–2023). Multiplex RT-PCR was used for HRV detection. Genotyping was conducted on 136 samples with Ct < 30 by sequencing the VP4/VP2 region. Species and genotype assignments were validated using BLAST and maximum-likelihood phylogenetic analysis (MEGA XII). HRV positivity increased from 16% (233/1404) before the pandemic to 60.4% (350/579) during the pandemic period. HRV-A and HRV-C predominated in both periods; HRV-C infection was significantly associated with severe pneumonia in the pre-pandemic period (OR 3.520; *p* = 0.012), but this association was not observed during the pandemic. A total of 72 genotypes were identified without a dominant type in either period. HRV circulation patterns shifted in the context of the COVID-19 pandemic, with a marked increase in prevalence. The high genotypic diversity observed across both periods underscores the importance of continuous molecular surveillance to better understand HRV circulation and its clinical relevance.

## 1. Introduction

Human rhinovirus (HRV) is the primary etiologic agent of upper respiratory tract infections (URIs) in children, generally considered mild, self-limiting, and benign conditions [1,2]. However, HRV has also been implicated in more severe outcomes, including exacerbations of chronic respiratory conditions such as asthma and COPD, as well as acute bronchitis, bronchiolitis, and community-acquired pneumonia in both pediatric and adult populations [3].

The clinical severity of HRV infections results from a complex interplay of host-related factors (e.g., age, comorbidities, immune status), environmental influences (e.g., seasonality, air pollution), and viral characteristics (e.g., species, genotype). These interactions can drive disease progression from mild URIs to serious lower respiratory tract involvement [1].

HRVs are non-enveloped, positive-sense single-stranded RNA viruses (ssRNA+) belonging to the *Picornaviridae* family, genus *Enterovirus*. Their genome spans approximately 7.2 to 7.5 kb and encodes a single polyprotein, which is processed into eleven proteins by viral proteases: four structural (VP1, VP2, VP3 and VP4) forming the capsid and seven non-structural proteins essential for replication and assembly [4].

Currently, HRVs are classified into three species: HRV-A, HRV-B, and HRV-C. HRV-A and HRV-B were originally defined through serotyping with neutralization tests, yielding 75 and 25 serotypes, respectively [5]. HRV-C, discovered in 2006, is genetically distinct and cannot be propagated in standard cell culture systems, which delayed its initial characterization [6].

The current classification of HRV relies on molecular genotyping, primarily through sequencing conserved genomic regions such as the 5′ untranslated region (5′-UTR), VP1, and VP4/VP2. To date, approximately 170 HRV genotypes have been identified: around 80 A, 32 B, and 57 C [7,8].

In Mexico, all three HRV species have been previously reported. Landa-Cardeña et al. in 2012 found HRV-A to be the most frequent in children with respiratory infections, followed by HRV-C and, less frequently, HRV-B [9]. A subsequent study by Aponte et al. in 2015 confirmed the predominance of HRV-A and HRV-C in both hospitalized and outpatient pediatric cases, linking them to mild and severe respiratory presentations [10].

HRV infections occur throughout the year, with peak incidence during autumn and spring [1]. During the COVID-19 pandemic, non-pharmaceutical interventions—including mask-wearing, lockdowns, and school closures—reduced the circulation of several respiratory viruses such as respiratory syncytial virus and adenovirus. However, HRV continued to circulate steadily, even coexisting with SARS-CoV-2 [11,12,13]. The clinical impact of this coinfection remains unclear: some studies have reported increased disease severity in patients coinfected with HRV and SARS-CoV-2 [12,14], whereas others found no significant effect of disease outcomes [15].

Recent studies from different regions have investigated HRV genotypic diversity and circulation patterns before and during the COVID-19 pandemic. For example, a study from Bulgaria reported that HRV-A and HRV-C were the predominant species, with HRV-A more frequent before the pandemic and HRV-C prevailing during the pandemic period [16]. Similarly, Fahmi Smaoui et al. (2025) showed that RV/EVs maintained high circulation throughout the COVID-19 pandemic in Tunisia, except during summer months, and that rhinovirus genotype diversity remained comparable to pre-pandemic levels [17]. However, data from Latin America remain scarce. Therefore, this study represents the first analysis of HRV genotypes in Mexico and contributes to the global understanding of HRV circulation and genetic diversity in the pandemic context.

Given the pandemic-induced shifts in respiratory virus circulation, we hypothesized that HRV genotypic distribution and diversity might also have been affected. Thus, the objective of this study was to compare HRV species distribution (HRV-A, HRV-B, HRV-C) and genotypic diversity in Mexican children diagnosed with pneumonia before and during the COVID-19 pandemic, to assess potential changes in viral circulation patterns.

## 2. Materials and Methods

### 2.1. Study Population

The study included two groups of clinical respiratory samples. The first group consisted of 1404 specimens collected between March 2010 and August 2013, of which 233 tested positive for rhinovirus/enterovirus. These samples were obtained from hospitalized children under five years of age diagnosed with pneumonia and treated at various medical institutions across Mexico: Hospital Central “Dr. Ignacio Morones Prieto” (San Luis Potosí), Hospital Regional Universitario IMSS Bienestar de Colima, Hospital General de Durango, Nuevo Hospital Civil de Guadalajara “Juan I. Menchaca,” Hospital Pediátrico de Coyoacán, Hospital General de Mexicali, Hospital Pediátrico, Centro Médico de Occidente IMSS, Hospital para el Niño de Toluca, Hospital General de México “Dr. Eduardo Liceaga,” Hospital de la Niñez Oaxaqueña, and Hospital Infantil de Tlaxcala.

The second group included 579 samples collected between 2021 and 2023 from children up to 5 years of age diagnosed with pneumonia. These samples were obtained from the following institutions: Hospital Regional de Alta Especialidad “Dr. Ignacio Morones Prieto” (San Luis Potosí), Antiguo Hospital Civil de Guadalajara “Fray Antonio Alcalde,” Hospital Pediátrico de Coyoacán, Hospital General de México “Dr. Eduardo Liceaga,” Hospital Municipal del Niño de Durango, Hospital Regional Universitario IMSS Bienestar de Colima and Hospital de las Culturas, San Cristóbal de las Casas, Chiapas.

The study was approved by the ethics and research committees of all participating institutions (Facultad de Medicina UNAM FM/DI/105/2020, Antiguo Hospital Civil de Guadalajara Fray Antonio Alcalde 052/20, Hospital Municipal del Niño de Durango 001/2021, Hospital Regional de Alta especialidad Dr Ignacio Morones Prieto 37-21, Hospital General de México Dr. Eduardo Liceaga DI/22/505/05/42, Hospital Pediátrico de Coyoacan 101-011-025-21, Hospital Regional Universitario de Colima CI 2021/02/CR/PED/130, Hospital de las Culturas, San Cristobal de las Casas, Chiapas). Parents or guardians of children with pneumonia were invited to have their children participate in the study. Written informed consent was obtained from parents or guardians of all participating children before any procedures were performed.

The first cohort represents a published study from 2010 to 2013 where the viral etiology of pneumonia in children younger than 5 years was determined [18]. The HRV positive samples from that study were used for genotyping.

For the second cohort, children who met the inclusion criteria who attended the emergency department or were hospitalized in the participating hospitals their parents were invited to have their children participate in the study. After informed consent was obtained, data and sample collection were carried out. Nasal swabs were collected from patients, placed in viral transport medium, and stored at −70 °C, before being sent to the Infectious Diseases Research Laboratory (LIEI), Faculty of Medicine, UNAM, where they were kept at −70 °C until further processing.

Children younger than 5 years with a clinical and/or radiological diagnosis of pneumonia were included. Pneumonia cases were defined by the presence of respiratory symptoms such as respiratory distress, cough, tachypnea, cyanosis, with or without fever within the first week of symptom onset, and/or a chest X-ray showing pulmonary infiltrates, classified as macro/micronodular, lobar, interstitial, multiple foci, pleural effusion, and mixed. Exclusion criteria included inadequate respiratory sample or lack of clinical and demographic data. Severe pneumonia was classified according to the World Health Organization (WHO) criteria [19]. All demographic and clinical characteristics were collected using a format designed specifically for this study across all the participating hospitals.

### 2.2. Respiratory Virus Detection and RNA Extraction

Pre-pandemic samples (2010–2013) were tested using the Anyplex™ RV16 multiplex RT-PCR assay (Seegene^®^, Seoul, Republic of Korea), targeting 16 respiratory viruses including HRV. Pandemic samples (2021–2023) were analyzed with the Allplex™ Respiratory Full Panel (Seegene^®^, Seoul, Republic of Korea), which detects an expanded range of respiratory pathogens. HRV positive samples were further analyzed and genotyped.

For HRV-positive samples, RNA was extracted from 140 µL of clinical material using the QIAamp Viral RNA Mini Kit (QIAGEN^®^, Hilden, Germany), following the manufacturer’s protocol.

### 2.3. cDNA Synthesis and PCR Amplification

Complementary DNA (cDNA) was synthesized using the ImProm-II™ Reverse Transcription System (Promega^®^ Madison, Wisconsin, USA) with random hexamers. Genotyping followed the protocol described by Wang Wei et al. (2015), involving an initial PCR targeting the VP4/VP2 region, followed by nested PCR [20]. The oligonucleotides used were:
**Primer****Sequence (5′–3′)****Position****Product Size**OSCCGGCCCCTGAATGYGGCTAA458667 bpOASACATRTTYTSNCCAAANAYDCCCAT1125ISACCRACTACTTTGGGTGTCCGTG547554 bpIASTCWGGHARYTTCCAMCACCANCC1087

OS outer sense, OAS outer antisense, IS inner sense, IAS inner antisense. The base positions of the primers were numbered according to the HRV-B serotype 14 genome (GenBank accession number NC_001490).

Nested PCR products were visualized on 1.5% agarose gels, purified using the QIAquick Gel Extraction Kit (QIAGEN^®^, Hilden, Germany), were quantified on the Thermo Scientific™ NanoDrop™ (Willmington, DE, USA) equipment and submitted to the DNA Synthesis and Sequencing Unit (USSDNA, IBT-UNAM) for Sanger sequencing.

### 2.4. Sequence Analysis and Genotyping

Samples with a cycle threshold (Ct) value greater than 30 consistently failed to amplify the VP4/VP2 region and were excluded from genotyping, similar cutoff values have also been reported in the literature for successful rhinovirus genotyping [16]. All samples with successful amplification were subjected to sequence analysis, even if they were excluded from the clinical comparison due to age.

Sequences were first analyzed via BLAST 2.16.0 against the GenBank database (NCBI) for preliminary genotype identification. Results were then validated using the RVdb rhinovirus typing tool (http://rvdb.mgc.ac.cn/rvdb/, accessed on 18 October 2024) [21,22].

Phylogenetic trees were constructed using MEGA XII software, aligning obtained sequences with known HRV references [23]. Trees were inferred via the maximum likelihood method under the GTR+G+I model with 1000 bootstrap replicates. Final tree visualization and editing were performed using Interactive Tree of Life (iTOL v7, https://itol.embl.de, accessed on 8 March 2025) [24]. The nucleotide sequences generated in this study are currently being processed for submission to the GenBank database. Accession numbers will be provided once the submission is completed.

### 2.5. Statistical Analysis

Statistical analyses were performed using IBM SPSS Statistics v25. Categorical variables (e.g., sex, hospitalization type, pneumonia severity) were compared using chi-square (χ^2^) or Fisher’s exact tests. Continuous variables (e.g., age, weight, height) were analyzed with the Mann–Whitney U test. Associations between clinical variables and severe pneumonia were evaluated with binary logistic regression. A *p*-value < 0.05 was considered statistically significant.

A total of 137 samples were included in the study, corresponding to 83 from the 2010–2013 period and 53 from 2021–2023. Among these, 53 samples were successfully amplified and genotyped in the VP4/VP2 region and were used for the comparative analysis by RV species. For the analysis of the study population, only patients under 5 years of age were considered. Variables with missing data were handled by listwise deletion, as SPSS excludes cases with incomplete information from each statistical test; therefore, percentages were calculated based on valid cases only.

## 3. Results

### 3.1. Frequency of Rhinovirus Detection

In the pre-pandemic group (2010–2013), a total of 1404 nasopharyngeal samples were analyzed from children with pneumonia under 5 years of age. HRV was detected in 233 samples, representing a positivity rate of 16%. In contrast, during the pandemic period (2021–2023), HRV was detected in 350 of 579 samples of children with pneumonia, indicating a significantly higher positivity rate of 60.4%, as previously reported [18,25]. Genotyping analysis was conducted in a subset of these previously reported HRV-positive samples.

### 3.2. HRV Species Distribution

**Figure 1:**
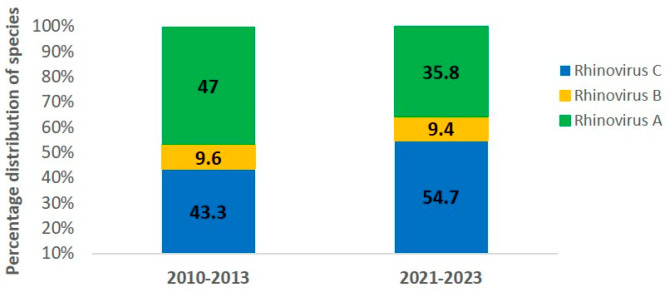
Percentage of human rhinovirus (HRV) species in pediatric pneumonia cases during the pre-pandemic (2010–2013) and pandemic (2021–2023) periods. HRV-A (*green*), HRV-B (*yellow*), and HRV-C (*blue*).

Analysis of species distribution showed that HRV-A was the most frequent in the pre-pandemic period, representing for 47.0% of the sequenced samples (39/83), followed by HRV-C (43.3%, 36/83) and HRV-B (9.6%, 8/83). During the pandemic, HRV-C became the most prevalent species (54.7%, 29/53), followed by HRV-A (35.8%, 19/53) and HRV-B (9.4%, 5/53). Despite these shifts, HRV-A and HRV-C remained the dominant species in both periods (see Figure 1).

### 3.3. Demographic and Clinical Characteristics

When comparing demographic and clinical parameters by RV species and study period, several notable differences were observed.

For rhinovirus A, 39 cases were identified in 2010–2013 and 16 in 2021–2023. No significant differences were observed in sex distribution or age between periods. However, children from the second period showed higher median height (0.79 vs. 0.68 m, *p* = 0.016) and weight (11.1 vs. 6.5 kg, *p* = 0.005) and a tendency of higher age. Clinically, respiratory rate was lower in the recent period (*p* = 0.024), with a trend toward lower body temperature. Most cases required hospitalization during the first period (97.4% vs. 75%, *p* = 0.028), but there were no significant differences in disease severity or radiographic pattern between periods. Regarding risk factors, no significant differences were observed (Table 1)

For rhinovirus B, 8 cases were detected in 2010–2013 and 5 in 2021–2023. There were no differences in the number of male and female patients infected with RV-B between periods. Patients from the second period were significantly older 11 (10) vs. 48 (38) months, *p* = 0.015) and presented greater height (*p* = 0.004) and weight (*p* = 0.004). No significant differences were found in clinical parameters, pneumonia severity, radiographic pattern, or any of the evaluated risk factors across periods (see Table 1)

In contrast, rhinovirus C, one of the most frequent species, showed significant differences between study periods. A total of 36 cases were detected in 2010–2013 and 25 in 2021–2023. Differences were observed in respiratory rate (*p* = 0.002), temperature (*p* = 0.016), thoracoabdominal dissociation (*p* = 0.016), as well as in disease severity and radiographic pattern. During 2021–2023, non-severe pneumonia became slightlly more common (52% vs. 48%, *p* = 0.145), while severe pneumonia predominated in 2010–2013 (66.7%). Although these differences were not statistically significant, the distribution suggests a shift toward a lower proportion of severe cases in the later period. Likewise, radiographic findings differed significantly (*p* = 0.003), with a predominance of the interstitial pattern in 2021–2023 (65%), compared to a more heterogeneous distribution in 2010–2013 (mainly interstitial and multifocal patterns). No significant differences were detected between periods for rhinovirus C in terms of co-infections, breastfeeding, immunocompromise, or vaccination status (see Table 1).

### 3.4. Risk Factors Associated with Severe Pneumonia

Potential risk factors for severe pneumonia were analyzed for both periods. During the 2010–2013 period, infection with rhinovirus C was significantly associated with the development of severe pneumonia, being observed in 55.8% of cases (OR = 3.520; 95% CI: 1.310–9.430; *p* = 0.012), in contrast to 50% (OR = 0.600; 95% CI: 0.169–2.130; *p* = 0.430) in the pandemic period. The following risk factors were also evaluated: coinfections, which were present in 65.1% of cases (OR = 0.508; 95% CI: 0.169–1.520; *p* = 0.228) vs. 54.2% (OR =1.105; 95% CI: 0.302–4.040; *p* = 0.880); immunocompromise status show 4.7% of cases (OR = 3.320; 95% CI: 0.263–41.950; *p* = 0.353) vs. 16.7% (OR = 4.979; 95% CI: 0.470–52.690; *p* = 0.182); absence of breastfeeding, reported in 48.8% (OR = 0.696; 95% CI: 0.263–1.830; *p* = 0.465) vs. 58.3% (OR =1.182; 95% CI: 0.333–4.195; *p* = 0.796); incomplete immunization schedule, observed in 60.5% of cases (OR = 1.170; 95% CI: 0.440–3.110; *p* = 0.752) vs. 37.5% (OR = 0.699; 95% CI: 0.169–2.890; *p* = 0.622); and absence of influenza vaccination, identified in 16.3% (OR = 0.423; 95% CI: 0.122–1.460; *p* = 0.175) vs. 20.8% (OR = 649; 95% CI: 0.135–3.127; *p* = 0.590). Although percentage differences were observed between groups, confidence intervals were wide and *p*-values did not reach statistical significance (Table 2).

### 3.5. Genotypic Diversity and Phylogenetic Analysis

Across both periods, 72 distinct HRV genotypes were identified. In 2010–2013, 28 HRV-A, 4 HRV-B, and 23 HRV-C genotypes were detected. In 2021–2023, the distribution included 16 HRV-A, 3 HRV-B, and 15 HRV-C genotypes (Table 3).

Between 2010–2013, RV-A80 and RV-A96 were each identified in three samples, while RV-B52 was detected in four. Other RV-B types, including B4 and B14, were also observed during this period. In the 2021–2023 period, RV-C1 and RV-C6 were the most frequently detected genotypes, present in five and four samples, respectively. Notably, several genotypes—such as A11, A12, A23, A28, A45, and A46—were identified only during the first period (2010–2013), whereas genotypes such as RV-C24 and RV-C40 appeared exclusively during the pandemic period (Table 3). These observations highlight the high genetic variability and co-circulation of multiple HRV genotypes in Mexican children with pneumonia and may suggest shifts in viral circulation dynamics associated with the pandemic.

**Figure 2:**
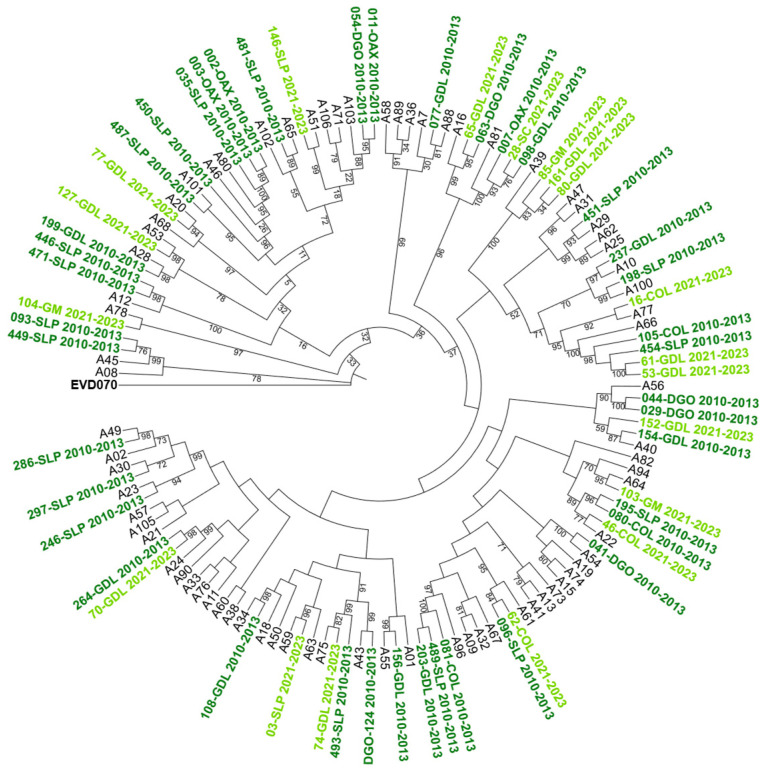
Phylogenetic tree of human rhinovirus A (HRV-A) based on the VP4/VP2 region. Constructed using the Maximum Likelihood (ML) method, GTR G+I model, with 1000 Bootstrap replicates. Only bootstrap values ≥ 70 are shown. Clinical isolates are indicated by sample ID numbers followed by hospital codes. Sequences from the 2010–2013 period are shown in dark green, and those from the 2021–2023 period in light green. Reference sequences are labeled by genotype number; GenBank accession numbers are listed in Supplementary Table S1. *Enterovirus EVD070* was used as an outgroup. Hospital codes: SLP = San Luis Potosí, COL = Colima, DGO = Durango, GDL = Guadalajara, OAX = Oaxaca, GM = Hospital General de México, SC = San Cristóbal.

**Figure 3:**
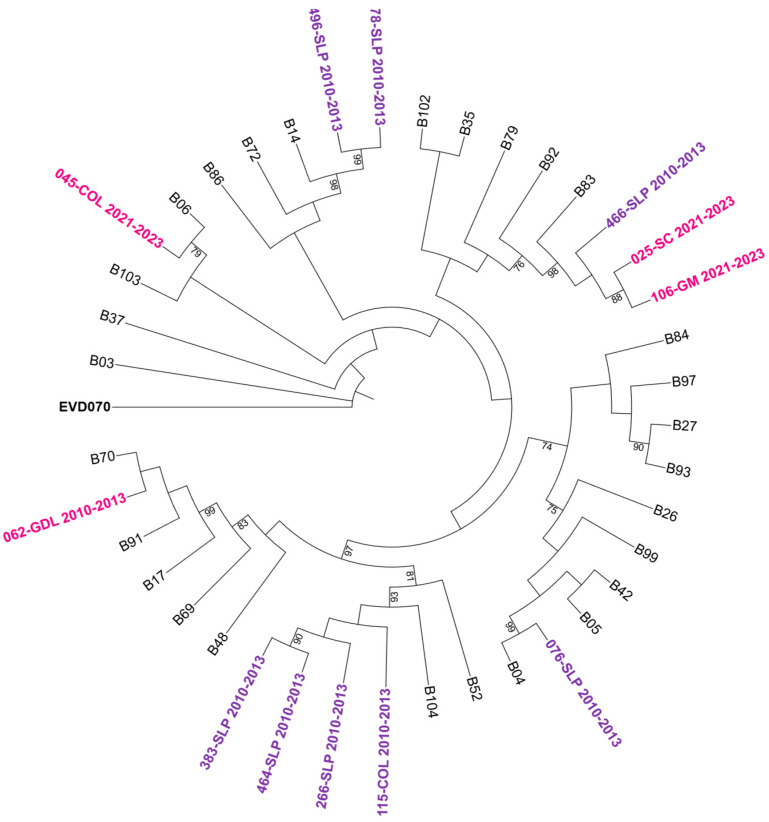
Phylogenetic tree of human rhinovirus B (HRV-B) based on the VP4/VP2 region. Constructed using the Maximum Likelihood (ML) method, GTR G+I model, with 1000 bootstrap replicates. Only bootstrap values ≥ 70 are shown. Sequences from the 2010–2013 period are represented in purple and those from 2021–2023 in bright pink. Reference sequences are labeled by genotype number; GenBank accession numbers are listed in Supplementary Table S1. *Enterovirus EVD070* was used as an outgroup. Hospital codes: SLP = San Luis Potosí, COL = Colima, GDL = Guadalajara, GM = Hospital General de México, SC = San Cristóbal.

**Figure 4:**
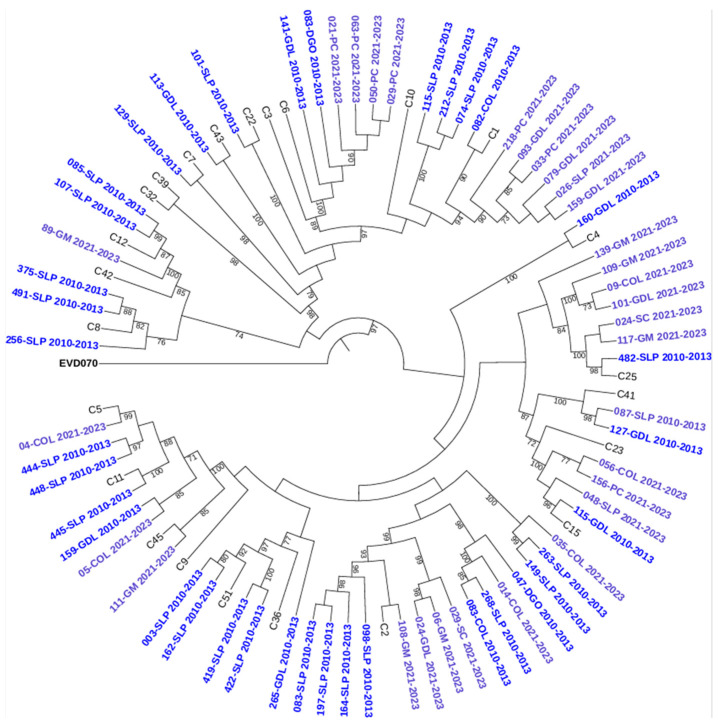
Phylogenetic tree of human rhinovirus C (HRV-C) based on the VP4/VP2 region. Constructed using the Maximum Likelihood (ML) method, GTR G+I method, with 1000 bootstrap replicates. Only bootstrap values ≥ 70 are shown. Sequences from 2010–2013 are indicated in dark blue and those from 2021–2023 in light blue. Reference sequences are labeled by genotype number; GenBank accession numbers are listed in Supplementary Table S1. *Enterovirus EVD070* was used as an outgroup. Hospital codes: SLP = San Luis Potosí, COL = Colima, DGO = Durango, GDL = Guadalajara, PC = Hospital Pediátrico de Coyoacán, GM = Hospital General de México, SC = San Cristóbal.

To further characterize the genetic relationships among the detected genotypes, maximum-likelihood phylogenetic trees were constructed based on VP4/VP2 nucleotide sequences. The trees confirmed the species-level classification and showed clustering of genotypes within each HRV species (see Figure 2, Figure 3 and Figure 4).

One sequence identified as 193-SLP from the 2021–2023 period, showed a p-distance of 0.313 (≈68.7% nucleotide identity) with its closest reference strain (HRV-B6) according to the Rhinovirus Genotype Database. This divergence largely exceeds the 12% inter-type threshold defined for RV-B [7]. Therefore, this sequence could not be assigned to any known genotype and was classified only at the species level as HRV-B.

## 4. Discussion

HRV is the second most frequently detected pathogen associated with pneumonia, with respiratory syncytial virus as the first [26], and asthma exacerbations in pediatric populations [2]. This study aimed to describe the distribution of HRV species and genotyping diversity in pediatric patients with pneumonia before and during COVID-19 pandemic in Mexico. Our findings revealed differences in species distribution and diversity between the two periods.

Prior to the COVID-19 pandemic, our research group reported a HRV positivity rate of 16% (233/1404) in children with pneumonia [18], which increased substantially to 60.4% (350/579) during the pandemic period [25]. This trend aligns with global reports indicating that HRV circulation remained stable despite pandemic restrictions [27,28]. HRV’s stability, environmental resilience, and high genomic diversity likely contributed to its sustained circulation [29,30].

Regarding HRV species, a shift in dominance was observed: HRV-A predominated before the pandemic, while HRV-C became more frequent during the pandemic. This pattern has been documented in various contexts. For example, Thongpan et al. (2021) reported an initial rise in pediatric respiratory infections due to HRV-A in early 2020, followed by a predominance of HRV-C [31]. Similarly, Georgieva et al. (2023) identified increased detection of HRV-C during the pandemic [16]. In Mexico, previous studies had also reported HRV-A and HRV-C as the most common species associated with upper and lower respiratory tract infections in children under five [10]. Although these shifts in species circulation are evident, none of the aforementioned studies—including the present one—found a consistent relationship between HRV species and clinical severity.

Children diagnosed during the pandemic were significantly older, taller, and heavier than those in the pre-pandemic period. This may reflect delayed exposure to respiratory viruses, including HRV, due to lockdowns and other pandemic-related social restrictions. A similar hypothesis has been proposed to explain delayed outbreaks of respiratory syncytial virus (RSV) in older infants during and after the pandemic [32], although further research is needed to confirm whether this pattern applies to HRVs.

In the first period (2010–2013), patients with HRV-C infection presented with severe pneumonia. In the second period there was a clear shift toward reduced clinical severity: lower respiratory rate, lower temperature, less thoraco-abdominal dissociation, and a higher frequency of non-severe pneumonia (Table 1). Although rhinovirus C has been primarily associated with wheezing and asthma exacerbations [33], both this study and previous reports have identified rhinovirus C infections in severe pneumonia cases [34,35]. The differences in HRV prevalence and species distribution as well as the overall clinical severity of pneumonia show some variation between the two periods.

The factors evaluated in this study—including rhinovirus C infection, the presence of comorbidities, incomplete vaccination, absence of breastfeeding, and viral coinfections—have previously been associated with increased severity in pediatric HRV infections [25]. Their inclusion in the present analysis allowed exploration of potential associations with the development of pneumonia and severe pneumonia across periods.

As observed, HRV-C infection was significantly associated with severe pneumonia during the pre-pandemic period (2010–2013), but this association was not evident in the pandemic cohort (2021–2023) (Table 2). This finding suggests that HRV-C played a more prominent role in pneumonia severity before the pandemic. Overall, the present findings support a role for HRV-C as an independent contributor to severe pneumonia in children, especially in settings with high viral diversity and frequent coinfections, as observed in the pre-pandemic period.

The genotype analysis did not reveal any predominant genotype in either of the evaluated periods; however, the results presented here may reflect a shift in HRV circulation; nevertheless, these findings should be interpreted with caution due to the small sample size. A genomic epidemiology study similarly reported that genotypes circulating in 2021 were replaced by different ones in 2022. The authors emphasized the importance of serological data to determine whether population-level immunity to genotypes prevalent in 2021 could have influenced genotype incidence in the following year, or whether these circulation changes reflected alternative seasonal patterns [36]. Another possibility is that the co-circulation of rhinovirus and SARS-CoV-2 may have affected HRV genetic diversity.

Seventeen of the genotypes detected in this study matched the most frequently reported types in a recent meta-analysis [1]. Among these, six genotypes (A22, A68, A88, A59, C43, and C45) have previously been associated with hospitalization. Although our findings do not establish a direct link between specific genotypes and clinical severity, this overlap suggests potential targets for future research into genotype-pathogenicity correlations.

Genotypic analysis identified 66 distinct HRV genotypes, confirming the high genetic diversity reported globally. While the VP4/VP2 region used here is reliable for species identification, one sample from 2021–2023 period could not be genotyped, likely due to high sequence divergence. Future studies might benefit from sequencing longer regions such as VP1 to improve genotyping resolution [7]. Together, these findings highlight the remarkable genetic diversity of circulating HRV strains and emphasize the need for continuous molecular surveillance to better understand their epidemiological and clinical relevance.

One limitation of the study was the absence of a comparison with healthy controls or asymptomatic individuals, which could have allowed a more accurate distinction between infection and colonization by rhinovirus. The study population included only children with pneumonia. A potential direction for future research could be to analyze the association between host immune status, viral load, and disease severity, as well as to assess the impact of co-infection with SARS-CoV-2 and HRV with a larger sample size.

## 5. Conclusions

This study documents a shift in HRV circulation among children with pneumonia before and during the SARS-CoV-2 pandemic, characterized by increased HRV positivity and a change in genotype distribution. Clinical severity differed between periods, revealing that HRV-C infection was associated with severe pneumonia primarily in the pre-pandemic years, when viral coinfections and inter-viral interactions appeared to play a key role in disease presentation. Genotypic diversity remained high across both periods, with no clear dominant pattern. Our findings underscore the importance of continuous monitoring of HRV genetic diversity and its clinical-epidemiological behavior, particularly in evolving post-COVID-19 scenarios.

## Figures and Tables

**Table 1:** Study population and clinical features of RV cases by species and study period.

	Rhinovirus A			Rhinovirus B			Rhinovirus C			Total	
	2010–2013 (n = 39)	2021–2023 (n = 16)	*p*	2010–2013 (n = 8)	2021–2023 (n = 5)	*p*	2010–2013 (n = 36)	2021–2023 (n = 25)	*p*	2010–2013 (n = 83)	2021–2023 (n = 46)
**Study population**											
Female, *n* (%)	10 (25.6)	7 (43.7)	0.300	4 (50)	1 (33.3)	0.576	7 (19.4)	10 (40)	0.061	21 (25.3)	18 (39.1)
Male, *n* (%)	29 (74.3)	9 (60)		4 (50)	4 (66.6)		29 (80.5)	15 (60)		62 (74.6)	28 (60.8)
Age (months), median (IQR)	9 (10)	12 (14)	0.126	11 (10)	48 (38)	**0.015**	12 (16)	12 (14)	0.726	11 (11)	12 (22)
Height (m), median (IQR)	0.68 (0.14)	0.79 (0.19)	**0.016**	0.53 (0.18)	1.0 (0.21)	**0.004**	0.73 (0.24)	0.74 (0.29)	0.594	0.69 (0.16)	0.81 (0.27)
Weight (kg), median (IQR)	6.5 (4.7)	11.1 (5.5)	**0.005**	4.5 (5.5)	14 (4.4)	**0.004**	8.6 (4.8)	7.45 (8.1)	0.489	7.5 (4.5)	10.1 (8.2)
**Hospital area**											
Ambulatory *n* (%)	1 (2.6)	3 (18.8)	**0.028**	0 (0)	1 (20)	0.151	2 (5.6)	0 (0)	0.334	3 (3.6%)	4 (8.7%)
Hospitalization *n* (%)	38 (97.4)	12 (75.0)		8 (100)	3 (60)		33 (91.6)	25 (100)		79 (95.1%)	40 (86.9%)
Intensive care unit *n* (%)	0	1 (6.7)		0 (0)	1 (60)		1 (2.8)	0 (0)		1 (1.2%)	2 (4.4%)
**Clinical characteristics**											
Respiratory rate (bpm), median (IQR)	44 (17)	35 (18)	**0.024**	60 (14)	26 (32)	0.101	46 (20)	37 (18)	**0.002**	46 (20)	35.5 (19)
Temperature (°C), median (IQR)	37 (1)	36.5 (1)	0.100	38.5 (2)	36.7 (2)	0.192	37 (1)	36.6 (1)	**0.016**	37 (1)	36.6 (1)
Cough, *n* (%)	33 (84.6)	14 (87.5)	1.00	7 (87.5)	4 (80)	1.00	33 (91.6)	20 (80)	0.254	73 (87.9)	38 (82.6)
Thoracoabdominal dissociation, *n* (%)	21 (53.8)	6 (37.5)	0.276	3 (37.5)	1 (20)	1.00	22 (61.1)	7 (28)	**0.016**	46 (55.4)	14 (30.4)
Intercostal retraction, *n* (%)	22 (56.4)	9 (56.2)	0.925	4 (50)	3 (60)	1.00	25 (69.4)	12 (48)	0.136	51 (61.4)	24 (52.1)
Xiphoid retraction, *n* (%)	10 (25.6)	7 (43.75)	0.155	2 (25)	2 (40)	0.576	7 (19.4)	6 (24)	0.587	19 (22.9)	15 (32.6)
Nasal flaring, *n* (%)	12 (30.7)	8 (50)	0.141	0 (0)	2 (40)	0.109	19 (52.7)	7 (28)	0.074	31 (37.3)	17 (37)
**Severity**											
Non-severe pneumonia, *n* (%)	23 (59)	7 (43.8)	0.303	5 (62.5)	2 (40)	0.592	12 (33.3)	13 (52)	0.145	40 (48.1)	22 (47.8)
Severe pneumonia, *n* (%)	16 (41)	9 (56.3)		3 (37.5)	3 (60)		24 (66.7)	12 (48)		43 (51.8)	24 (52.1)
**Radiographic pattern**											
Micro and/or macronodular, *n* (%)	5 (12.8)	2 (12.5)	0.142	1 (12.5)	2 (40)	0.511	6 (16.6)	5 (20)	**0.008**	12 (14.4)	9 (19.5)
Multiple foci, *n* (%)	5 (12.8)	0 (0)		1 (12.5)	0 (0)		12 (33.3)	0 (0)		18 (21.7)	0 (0.0)
Lobar, *n* (%)	0 (0)	1 (6.25)		1 (12.5)	1 (20)		4 (11.1)	2 (8)		5 (6.02)	4 (8.6)
Interstitial, *n* (%)	17 (43.5)	10 (62.5)		1 (12.5)	0 (0)		9 (25.0)	13 (52)		27 (32.5)	22 (47.8)
Mixed, *n* (%)	4 (10.2)	0 (0)		1 (12.5)	0 (0)		3 (8.3)	0 (0)		8 (9.6)	0 (0.0)
Pleural effusion, *n* (%)	NA	NA		0 (0)	1 (20)		NA	NA		0 (0.0)	1 (2.1)
NA	8 (20.5)	3 (18.7)		3 (37.5)	1 (20)		2 (5.5)	5 (20)		13 (15.6)	10 (21.7)
**Comorbidities and risk factors**											
Coinfections, *n* (%)	29 (74.3)	10 (62.5)	0.515	6 (75)	2 (40)	0.293	25 (69.4)	13 (52)	0.167	60 (72.2)	25 (54.3)
Immunocompromise, *n* (%)	2 (5.1)	2 (12.5)	0.571	0	0		1 (2.8)	3 (12)	0.296	3 (3.6)	5 (10.8)
Breastfeeding absence, *n* (%)	20 (51.3)	11 (68.8)	0.235	4 (50)	3 (60)	1.00	20 (55.6)	12 (48)	0.561	44 (53.0)	26 (56.5)
Incomplete vaccination schedule, *n* (%)	20 (51.3)	7 (43.8)	0.612	4 (50)	2 (40)	1.00	24 (66.7)	11 (44)	0.078	48 (57.8)	20 (43.4)
Influenza vaccine absence, *n* (%)	6 (15.4)	4 (25)	0.453	0 (0)	2 (40)	0.128	10 (27.8)	6 (24)	0.741	16 (19.3)	12 (26.0)

Chi-square test: sex, cough, thoracoabdominal dissociation, intercostal retraction, nasal flaring, disease severity, coinfections, incomplete vaccination schedule, and absence of influenza vaccination. Fisher’s exact test: hospital area, radiographic pattern, immunocompromised status, and absence of breastfeeding. Mann–Whitney U test: age, height, weight, respiratory rate, and temperature. Additionally, a small number of patients had incomplete demographic or clinical data (e.g., missing sex, height, or weight). These cases were retained in the analysis, and the total number of valid observations is indicated for each variable in the revised table. Values in bold indicate statistical significance.

**Table 2:** Risk factors associated with severe pneumonia in children infected with rhinovirus, during the pre-pandemic and pandemic period.

Risk Factors	2010–2013 (*n =* 83)				2021–2023 (*n =* 46)			
	Non-Severe Pneumonia n = 40 n (%)	Severe Pneumonia n = 43 n (%)	OR (95% CI)	*p*	Non-Severe Pneumonia n = 22 n (%)	Severe Pneumonia n = 24 n (%)	OR (95% CI)	*p*
Rhinovirus C infection	12 (30)	24 (55.8)	3.520 (1.310–9.430)	0.012	13 (59.1)	12 (50)	0.600 (0.169–2.130)	0.430
Coinfections	32 (80)	28 (65.1)	0.508 (0.169–1.520)	0.228	12 (54.5)	13 (54.2)	1.105 (0.302–4.040)	0.880
Immunocompromise	1 (2.5)	2 (4.7)	3.320 (0.263–41.950)	0.353	1 (4.54)	4 (16.7)	4.979 (0.470–52.690)	0.182
Breastfeeding absence	23 (57.5)	21 (48.8)	0.696 (0.263–1.830)	0.465	12 (54.5)	14 (58.3)	1.182 (0.333–4.195)	0.796
Incomplete vaccination schedule	22(55)	26 (60.5)	1.170 (0.440–3.110)	0.752	11 (50)	9 (37.5)	0.699 (0.169–2.890)	0.622
Influenza vaccine absence	9 (22.5)	7 (16.3)	0.423 (0.122–1.460)	0.175	7 (31.8)	5 (20.8)	0.649 (0.135–3.127)	0.590

The distribution of clinical and demographic risk factors among children with non-severe and severe pneumonia in each period is described. Odds ratios (ORs) and 95% confidence intervals (CIs) were calculated to assess associations.

**Table 3:** Distribution of HRV genotypes in Mexico during the pre-pandemic and pandemic period.

	RV-A			RV-B			RV-C	
	Pre-Pandemic	Pandemic		Pre-Pandemic	Pandemic		Pre-Pandemic	Pandemic
A10	1	--	B4	1	--	C1	1	6
A12	2	--	B6	--	1	C2	4	1
A16	1	1	B14	2	--	C5	--	1
A20	--	1	B52	4	--	C6	2	4
A22	2	1	B70	--	1	C11	1	--
A23	1	--	B83	1	2	C12	2	1
A24	1	1				C15	1	3
A28	1	--				C17	1	--
A30	1	--				C19	--	1
A34	1	--				C21	1	--
A39	--	3				C22	1	--
A40	1	--				C24	--	2
A43	1	--				C25	--	2
A44	1	--				C27	1	1
A45	2	--				C28	1	--
A49	1	--				C29	--	1
A51	--	1				C33	3	1
A53	--	1				C35	2	1
A55	1	--				C37	1	
A56	2	--				C38	2	--
A59	---	1				C40	--	3
A61	1	1				C41	2	--
A64	--	1				C43	1	
A65	1	--				C49	2	
A66	2	2				C50	1	--
A75	1	1				C51	1	--
A77	--	1				C56	1	--
A78	--	1				C57	3	
A80	3	--				C59	1	1
A81	2	1						
A85	--	1						
A88	1	--						
A96	3	--						
A100	1	--						
A101	1	--						
A103	2	--						
A108	1							
Total	39	19		8	4		36	29

The distribution of human rhinovirus (HRV) genotypes detected in clinical samples during the pre-pandemic (2010–2013) and pandemic (2021–2023) periods, categorized by species (HRV-A, HRV-B, HRV-C) are shown. Each cell indicates the number of samples in which a given genotype was identified.

## Data Availability

The data that support the findings of this study are available from the corresponding author upon reasonable request.

## Supplementary Materials

The following supporting information can be downloaded at: https://www.mdpi.com/article/10.3390/pathogens14121236/s1, Table S1: The list of reference sequences in this study.
