# Supplementary material for: Genotypic Diversity of Human Rhinovirus in Children with Pneumonia Before and During the COVID-19 Pandemic in Mexico
Source: Pathogens. 2025 Dec 4;14(12):1236. doi: 10.3390/pathogens14121236 (PMC12736054; doi:10.3390/pathogens14121236)
Supplement: Supplementary file 1 [file pathogens-14-01236-s001.zip › pathogens-3988763-supplementary.pdf]

Supplementary Table 1. The list of reference sequences in this study.

Genotypes/serotypes	Accession number	Genotypes/serotypes	Accession number	Genotypes/serotypes	Accession number
A001	FJ445111.1	A045	FJ445132.1	A088	DQ473504.1
A002	X02316.1	A046	DQ473506.1	A089	FJ445184.1
A007	FJ445176.1	A047	FJ445133.1	A090	FJ445167.1
A008	FJ445113.1	A049	DQ473496.1	A094	FJ445185.1
A009	FJ445114.1	A050	FJ445135.1	A096	FJ445171.1
A010	FJ445178.1	A051	FJ445136.1	A100	FJ445175.1
A011	EF173414.1	A053	DQ473507.1	A101	GQ415051.1
A012	EF173415.1	A054	FJ445138.1	A102	EF155421.1
A013	FJ445116.1	A055	DQ473511.1	A103	JQ747749.1
A015	DQ473493.1	A056	FJ445140.1	A105	JN614995.1
A016	JN562722.1	A057	FJ445141.1	A106	JX025555.1
A018	FJ445118.1	A058	FJ445142.1		
A019	FJ445119.1	A059	DQ473500.1	B003	DQ473485.1
A020	FJ445120.1	A060	FJ445143.1	B004	DQ473490.1
A021	FJ445121.1	A061	FJ445144.1	B005	FJ445112.1
A022	FJ445122.1	A062	FJ445145.1	B006	DQ473486.1
A023	DQ473497.1	A063	FJ445146.1	B014	K02121.1
A024	FJ445190.1	A064	FJ445181.1	B017	EF173420.1
A025	FJ445123.1	A065	FJ445147.1	B026	FJ445124.1
A028	DQ473508.1	A066	FJ445148.1	B027	FJ445186.1
A029	FJ445125.1	A067	FJ445149.1	B035	FJ445187.1
A030	FJ445179.1	A068	FJ445150.1	B037	EF173423.1
A031	FJ445126.1	A071	FJ445152.1	B042	FJ445130.1
A032	FJ445127.1	A073	DQ473492.1	B048	DQ473488.1
A033	FJ445128.1	A074	DQ473494.1	B052	FJ445188.1
A034	FJ445189.1	A075	DQ473510.1	B069	FJ445151.1
A036	DQ473505.1	A076	FJ445182.1	B070	DQ473489.1
A038	FJ445180.1	A077	FJ445154.1	B072	FJ445153.1
A039	AY751783.1	A078	EF173418.1	B079	FJ445155.1
A040	FJ445129.1	A080	FJ445156.1	B083	FJ445161.1
A041	DQ473491.1	A081	FJ445157.1	B084	FJ445162.1
A043	FJ445131.1	A082	FJ445160.1	B086	FJ445164.1

Genotypes/serotypes	Accession number	Genotypes/serotypes	Accession number
B091	FJ445168.1	C043	JX074056.1
B092	FJ445169.1	C045	JN837686.2
B093	EF173425.1	C051	JF317015.1
B097	FJ445172.1		
B099	FJ445174.1	EVD070	D00820.1
B102	JX074053.1		
B103	JN798572.1		
B104	FJ445137.1		
C001	EF077279.1		
C002	JQ245968.2		
C003	EF186077.2		
C004	EF582385.1		
C005	EF582386.1		
C006	EF582387.1		
C007	DQ875932.2		
C008	GQ223227.1		
C009	GQ223228.1		
C010	GQ323774.1		
C012	JF317017.1		
C015	GU219984.1		
C022	JN621242.1		
C023	KJ675506.1		
C025	JF317013.1		
C032	JN798581.1		
C036	JN541267.1		
C039	JN205461.1		
C041	JN798565.1		
C042	JQ994500.1		
